# Muscle-MRI and Functional Levels for the Evaluation of Upper Limbs in Duchenne Muscular Dystrophy: A Critical Review of the Literature

**DOI:** 10.3390/medicina58030440

**Published:** 2022-03-17

**Authors:** Lara Cristiano, Claudia Brogna, Giorgio Tasca, Tommaso Verdolotti, Marika Pane, Eugenio Mercuri

**Affiliations:** 1 Pediatric Neurology Unit, Fondazione Policlinico Universitario “A. Gemelli”, IRCCS, 00168 Rome, Italy; laracristiano2811@gmail.com (L.C.); marika.pane@policlinicogemelli.it (M.P.); eugeniomaria.mercuri@policlinicogemelli.it (E.M.); 2 Nemo Clinical Centre, Fondazione Policlinico Universitario “A. Gemelli”, IRCCS, 00168 Rome, Italy; 3 Unità Operativa Complessa di Neurologia, Fondazione Policlinico Universitario “A. Gemelli”, IRCCS, 00168 Rome, Italy; giorgio.tasca@policlinicogemelli.it; 4 Institute of Radiology, Fondazione Policlinico Universitario “A. Gemelli”, IRCCS, 00168 Rome, Italy; tommaso.verdolotti@policlinicogemelli.it; 5 Pediatric Neurology, Università Cattolica del Sacro Cuore, 00168 Rome, Italy

**Keywords:** muscle MRI, PUL, DMD, upper limbs

## Abstract

Many qualitative and quantitative Magnetic Resonance Imaging (MRI) techniques have been applied to evaluate muscle fat degeneration in Duchenne muscular dystrophy (DMD) subjects, but only few studies have focused on the upper limbs. We reviewed the literature in order to evaluate the association between muscle MRI findings and motor function levels in the upper limbs of DMD patients. Ten studies with upper limb muscle MRI data were available. Four explored all upper limb segments, while six explored only the forearm. Functional assessments were performed in nine of the ten studies. All of the studies showed a significant correlation between muscle MRI changes and motor function levels in both ambulant and non-ambulant DMD patients.

## 1. Introduction

Duchenne Muscular Dystrophy (DMD) is an X-linked, progressive, neuromuscular disorder, affecting approximately 1/3500–6000 live male births, caused by the absence of a functional dystrophin protein, leading to progressive muscle degeneration and to a pattern of loss of specific functional milestones. Over the last few years, new experimental therapies developed for the treatment of DMD have highlighted the need for non-invasive objective diagnostic biomarkers in order to assess the efficacy of the new therapeutic approaches during the different stages of the disease [1,2,3,4,5,6]. 

Magnetic Resonance Imaging (MRI) and spectroscopy (MRS) have proven to be sensitive and reproducible markers of muscle damage and disease progression in both ambulant and non-ambulant DMD patients and are being used to evaluate therapeutic responses across all stages of the disease, as well as an endpoint in clinical trials [7,8,9,10]. It has also been shown that the same pattern of muscle can be observed in Becker muscular Dystrophy and female carriers of dystrophinopathy, even if with an overall lesser extent [11,12]. 

Different MRI qualitative (using standard T1 and T2 sequences) and quantitative (using Dixon sequences) techniques have been used to evaluate muscle involvement and the gradient of disease progression in DMD subjects by identifying fatty infiltration and muscle edema [13,14,15,16,17,18,19,20,21,22,23]. In addition, in the last few years, MRI spectroscopy has given important information on the metabolic composition of the muscular structure [7,8,14,15,18,20].

Muscle MRI has been widely used to study the lower limbs of DMD patients [8,9,10], but less has been reported on its use for the evaluation of the upper limbs [14,15,16,17,18,19,20]. 

The majority of the studies reporting upper limb muscle MRI have focused on distal muscles, therefore not taking into account proximal changes that may occur at an earlier stage of the disease, which is in agreement with the well-known proximal to distal progression. This is particularly relevant in patients who are ambulant or are about to lose ambulation and still rely on upper extremities for most of their daily life activities. This has recently become even more important at the time these patients are considered to be included in clinical trials and a combined approach using clinical functional scales and MRI could provide accurate information on the progression of the disease and the possible efficacy of the intervention.

The aim of this review was to evaluate the existing literature on upper limb muscle MRI in both ambulant and non-ambulant DMD patients and, when available, the correlation between muscle MRI and motor functional levels. 

## 2. Material and Methods

We considered the studies published as full-text articles in indexed journals, which investigated the association between muscle MRI and motor functional levels in the upper limbs of both ambulant and non-ambulant DMD patients. Only articles written in English with an available abstract were included. No publication date limits were set. Expert opinions, case reports, letters to the editor, unpublished reports, reviews of the literature, abstracts from scientific meetings, and book chapters were excluded from the present review.

Scopus, Cochrane Library MEDLINE via PubMed, and Embase were searched using the keywords: “Duchenne muscular dystrophy”, “muscle magnetic resonance imaging”, “muscle MRI”, “functional levels”, “upper limb MRI”, and their MeSH terms in any possible combination. 

**Figure 1:**
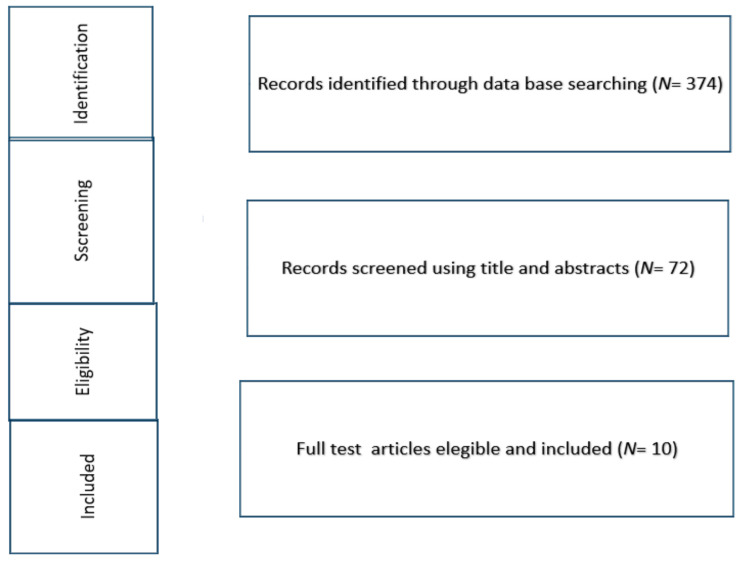
Flow chart that shows the selection of the papers.

The reference lists of relevant studies were screened to identify other studies of interest. The search was reiterated until 15 November 2021. Three hundred seventy- four records were identified (Figure 1).

## 3. Results

After screening 72 articles by title and abstract, 10 studies met the inclusion criteria and were selected: five were prospective cross-sectional studies [14,15,16,17,18] and five were prospective longitudinal studies (Table 1) [19,20,21,22,23]. These included information on a total of 296 DMD patients. The mean age was 11.5 ± 1.71 SD years. One hundred sixty-two DMD patients were ambulant and 140 were non-ambulant. In eight studies [14,16,17,18,19,21,22,23], patients were under steroid treatment. In two studies, the treatment was not specified [15,20].

Three studies reported information related to the underlying genetic mutation in eighty-nine patients (29% of the overall cohort) who carried deletions of specific exons [14,20,22]. A mean follow-up of at least 12 months was reported in five out of ten studies (50% of the overall cohort).

Of the 10 studies using MRI, four examined the entire upper limb (shoulder, arm, and forearm) [15,16,18,23], and six assessed the forearm only [14,17,19,22]. Different MRI techniques were used for qualitative or quantitative assessment of fat degeneration in muscles. Only three studies, accounting for a total of 31 patients, used a semi-quantitative evaluation of fatty infiltration Turbo spin echo (TSE) T1 sequences evaluated by Mercuri score) [16,17,23], three studies [19,21,22] used quantitative fat-fraction analysis, and four studies [14,15,18,20] used both quantitative measurements and MRS. 

Since the aim of this study was to analyze individual papers, a descriptive analysis was used, including information according to the type of the study (prospective or longitudinal), the type of the MRI technique used (quantitative or semiquantitative), and according to the type of motor functional tests used. 

### 3.1. Muscle MRI and Functional Measures 

Functional measures were available in nine of the ten studies [15,16,17,18,19,20,21,22,23] (Table 1).

Seven of the nine studies [15,16,17,18,19,21,23] used the performance of upper limb (PUL) test. Three of the nine studies also used the Brooke Upper Extremity Scale [15,18,22] and four also used grip and pinch strength [15,18,20,22]. Two studies [20,22] used the Motor Function Measure (MFM) and the Movie Plate assessment. The analysis of MRI protocols showed that seven studies used quantitative MRI evaluation [14,15,18,19,20,21,22], whereas three studies used qualitative evaluation [16,17,23]. 

In all of the studies, a significant correlation between muscle involvement assessed by MRI and functional tests was found. 

### 3.2. Cross-Sectional Studies 

Three of the five cross-sectional studies used quantitative MRI and MRS [14,15,18], while the remaining two implemented a semiquantitative visual assessment of T1 sequences [16,17]. 

Two of the five studies only assessed the forearm [14,17], while three assessed all three domains including the shoulder, arm, and forearm [15,16,18].

These latter studies showed the highest rate of involvement in the shoulder muscles, followed by the upper arm, and the forearm muscles [15,16,18]. Two of the three studies [15,18] compared muscle MRI findings in DMD to controls, showing that MRS-T2 and quantitative T2 measurements were higher in participants with DMD when compared to controls. 

All three studies also reported functional assessments [15,16,18]. MRI-T2 and fat fraction (FF) were found to have a strong correlation with grip strength, with the Brooke Upper Extremity Scale (*p* = 0.001) and with the PUL [15]. In one study [18] T2, FF, and proton magnetic resonance spectroscopy (1 H MRS) fat fraction measures of the upper extremity muscles were correlated with the total PUL and the proximal and mid-level PUL (*p* = 0.001), as well as to the distal PUL (*p* = 0.013) and the strength tests. Forbes and colleagues [18] also found a correlation between a composite of all upper extremity muscles examined with MRI (i.e., average of deltoid, triceps brachii, biceps brachii, anterior forearm, and posterior forearm) and the total PUL, in both ambulant (*p* = 0.003) and non-ambulant (*p* = 0.01) patients. In another study [16], the semiquantitative scores of all domains were also correlated with the total PUL score, providing details between the degree of MRI involvement and PUL thresholds in each segment. A diffuse and severe fatty replacement of all muscles at the shoulder level was found in all patients with a PUL shoulder functional score less than five; at mid-level, some degree of involvement could already be detected in patients with scores on the PUL mid domain less than six. At the distal level, diffuse and severe involvement was found only in patients who had very low scores (eight or below) on the PUL distal domain.

The two studies exploring the forearm only [14,17] reported that flexor muscles had a higher fat infiltration than extensor muscle groups on T2 sequences. No functional tests were used. 

Finally, in a small case series focusing on possible early involvement of distal muscles [17], a selective abnormal signal on T1 sequences in the supinator muscle at the forearm level was found in all patients, including those with no or little proximal involvement. No formal functional assessment was reported, but the distal changes were associated with an inability to perform a full supination of the forearm, with less than 75% of the predicted range of movement.

### 3.3. Longitudinal Prospective Studies

Four of the five studies used quantitative MRI [19,20,21,22]; in two of these studies, muscle cross sectional area was also evaluated [19,22], and one also used proton spectroscopy [20]. The last study used T1 sequences with semiquantitative visual assessment [23].

Four of the five assessed the forearm only [19,20,21,22], while the other explored all three domains including the shoulder, arm, and forearm [23]. Three studies had a follow-up of 1 year [19,23], one of 2 years [20], and one of 3 years [21]. Two of the studies reported details of muscle MRI findings in DMD patients carrying a specific group of deletions amenable to skip individual exons [20,22]. 

All five studies showed an increase of muscle impairment on MRI over time, and all reported a positive association with functional assessments [19,20,21,22,23]. Hogrel and colleagues [20] described a significant correlation between FF of the forearm muscles and a functional test including MyoGrip, MyoPinch, MoviPlate, and MFM-Total score at baseline (*p* = <0.001) in both ambulant and non-ambulant patients. MRI changes correlated with MoviPlate performance in ambulant patients and with grip strength in non-ambulant patients. 

Naarding and colleagues [21] reported that the mean annual increase in elbow flexor FF in the forearm of 20 non-ambulant patients predicted loss of hand-to-mouth movement independently of age. Lillien and colleagues [22] reported a progressive increase of FF in flexors forearm at 12, 24, and 36 months and in extensor muscles at 24 and 36 months. They also reported a correlation between FF of flexors and extensors, as well as grip and pinch strength, and total MFM score.

The study assessing all domains with a semiquantitative assessment [23] showed increased abnormalities on all domains on muscle MRI and a significant correlation between MRI changes and PUL changes at the shoulder level (*p* = 0.01). 

## 4. Discussion

Our review confirms the heterogeneity in terms of study design, sequences used, and choice of segments scanned (whole upper limb versus forearm) in the existing literature.

Overall, these results highlight the importance of assessing multiple domains. Even if there is a clear proximal to distal gradient, the changes do not always occur sequentially, as distal MRI changes can already be detected when proximal muscles are still relatively spared [17]. 

Six of the 10 papers [14,17,19,20,21,22] only reported information on the forearm. However, this domain is more diffusely involved in non-ambulant patients, generally at the end of the second decade. Since most forearm muscles are relatively spared in ambulant patients or in those who have just lost ambulation, this segment may not be the ideal candidate for studies in these subgroups for whom the assessment of a more proximal segment may provide additional important information. 

The review also suggests that muscle MRI findings are often associated with functional impairment. It is of note that, irrespective of the protocol or sequences used, a significant correlation was always found between both qualitative and quantitative muscle MRI and functional assessments [14,15,19,20,21,22]. The association was stronger at baseline, while it was less striking when comparing imaging and functional changes over time. This may be due to several factors. While functional scores reflect the activity of one muscle or even groups of muscles, MRI measurements are often more limited. Several studies have compared a single domain on MRIs of upper limbs, mainly the forearm, to various functional aspects, that in some cases, involved different upper limb domains, such as total score on the PUL. Not surprisingly, in these studies, the correlation with general scales was poorer than with pinch grip or other distal activities more directly related to forearm muscles. 

Furthermore, in studies using quantitative assessments, only a limited number of slices were analysed for each segment and these may be not representative of the overall extent of impairment of the muscle or muscle groups. 

Another possibility is that muscle MRI changes may precede functional changes. Little has been reported about the possible prognostic value of MRI in specific muscles or groups of muscles to predict later functional changes [23]. This was only partly explored in a longitudinal study using visual analysis, but these results should be confirmed using a more quantitative approach [23].

## 5. Conclusions

In conclusion, all the available papers provide some relevant information on the use of different sequences and the choice of upper limb segments, both in clinical practice and in a research setting. Due to the heterogeneity in sequences and cohorts, as well as the limited number of patients studied, a number of questions regarding which protocol should be used in different clinical stages still remain. Similarly, more work is also needed to establish the extent of upper limb changes on MRI over time in all domains, as well as if and how these can predict functional changes. 

## Figures and Tables

**Table 1:** List of prospective and longitudinal studies included.

Authors	Study Type	Genotype	Patients	Mean Age (Age in Years + SD)	Non-Ambulant Patients	Upper Limb Section Evaluated	MRI Type and Scoring	Motor Functional Tools
Prospective cross sectional studies								
**Wary et al., 2015 [14]**	Prospective cross-sectional	53 skippable patients	24	11.2 ± 3.7	14	Forearm	quantitative FF and T2 MRI, 31P NMRS (3.0-T MRI system)	None
**Willcocks et al., 2016 [15]**	Prospective Cross-sectional	Not reported	22	10.8 ± 2.5	2	Shoulder, upper arm, forearm	quantitative FF and T2 MRI 1 H-MRS, (3.0-T MRI system)	PUL, Brooke Upper Extremity Scale, grip and pinch strength
**Brogna et al., 2018 [16]**	Prospective Cross-sectional	Not reported	31	12.7 ± 5.5	14	Shoulder, arm, forearm	T1 MRI Mercuri Score (1.5-T MRI system)	PUL
**Tartaglione et al., 2018 [17]**	Case series	Not reported	4	5–15	1	Forearm	T1 MRI Mercuri Score (1.5-T MRI system)	Distal PUL
**Forbes et al., 2020 [18]**	prospective cross-sectional	Not reported	119	12 ± 3	35	Shoulder, arm, forearm	Quantitative FF and T2 MRI 1 H MRS (3.0-T MRI system)	PUL, Brooke upper extremity Scale, Grip Strength, Pinch Stength
**Prospective longitudinal studies**								
**Ricotti et al., 2016 [19]**	Prospective longitudinal	Not reported	15	13.2	15	Forearm	quantitative MRI FF and T2 (3.0-T MRI system)	PUL, Myopinch, Myogrip, Moviplate, Egen Klassification (EK2)
**Hogrel et al., 2016 [20]**	Prospective longitudinal	53-skippable patients	25	8.2 in ambulant, 13.9 in non ambulant patients	15	Forearm	quantitative MRI FF and T2 and phosphorous MRS (3.0-T MRI system)	MFM, hand grip and key pinch strength, MoviPlate
**Naarding et al., 2021 [21]**	Prospective longitudinal	Not reported	20	13.5 (12.5–16.4)	20	Forearm	quantitative MRI FF (3.0-T MRI system)	PUL
**Lillien et al., 2021 [22]**	Prospective longitudinal	53-skippable patients	40	11.7 (3.4)	22	Forearm	quantitative MRI FF and cross-sectional area (1.5 and 3.0-T MRI system)	Brooke score, MFM, hand grip and key pinch strength, and MoviPlate
**Brogna et al., 2021 [23]**	Prospective longitudinal	Not reported	27	5–30	17	shoulder arm, forearm	T1 MRI Mercuri Score (1.5-T MRI system)	PUL

## Data Availability

Data is contained within the article.
